# Crystal structure of 4′-(2-meth­oxy­quinolin-3-yl)-1′-methyl­dispiro­[indan-2,2′-pyrrolidine-3′,3′′-indoline]-1,3,2′′-trione

**DOI:** 10.1107/S2056989015023026

**Published:** 2015-12-12

**Authors:** Sadasivam Mathusalini, Vijayan Viswanathan, Palathurai Subramaniam Mohan, Chia-Her Lin, Devadasan Velmurugan

**Affiliations:** a Department of Chemistry, School of Chemical Sciences, Bharathiar University, Coimbatore 641 046, India; b Centre of Advanced Study in Crystallography and Biophysics, University of Madras, Guindy Campus, Chennai 600 025, India; c Department of Chemistry, Chung Yuan Christian University, Chung-Li 32023, Taiwan

**Keywords:** crystal structure, spiro-indane, spiro-indolino, quinoline, pyrrolidine, hydrogen bonding

## Abstract

In the title compound, C_30_H_23_N_3_O_4_, the central 1-methyl­pyrrolidine ring adopts a twist conformation on the N—CH_2_ bond. The pyrrolidin-2-one ring of the indolin-2-one ring system also has a twist conformation on the C—C bond involving the spiro C atom and the carbonyl C atom. The five-membered ring of the indene-1,3-dione moiety has an envelope conformation with the spiro C atom as the flap. The quinoline ring system adopts an almost planar conformation (r.m.s. deviation = 0.04 Å). The mean planes of the indolin-2-one ring system, the indene-1,3-dione ring system and the the quinoline ring system are inclined to the mean plane of the central 1-methyl­pyrrolidine ring by 77.97 (7), 86.98 (7) and 46.58 (6)°, respectively. In the crystal, mol­ecules are linked *via* N—H⋯N hydrogen bonds, forming chains along the *b* axis. The chains are linked *via* a number of C—H⋯O hydrogen bonds, and C—H⋯π and π–π inter­actions [inter-centroid distance = 3.7404 (9) Å], forming a three-dimensional network.

## Related literature   

For the biological activity of pyrrolidine and indole derivatives, see: Babu *et al.* (2012 ▸); Savithri *et al.* (2014 ▸); Govind *et al.* (2003 ▸); Gayathri *et al.* (2005 ▸); Li *et al.* (2004 ▸); Bellina & Rossi (2006 ▸). For the crystal structure of a similar di­spiro­indoline compound, see: Nirmala *et al.* (2009 ▸).
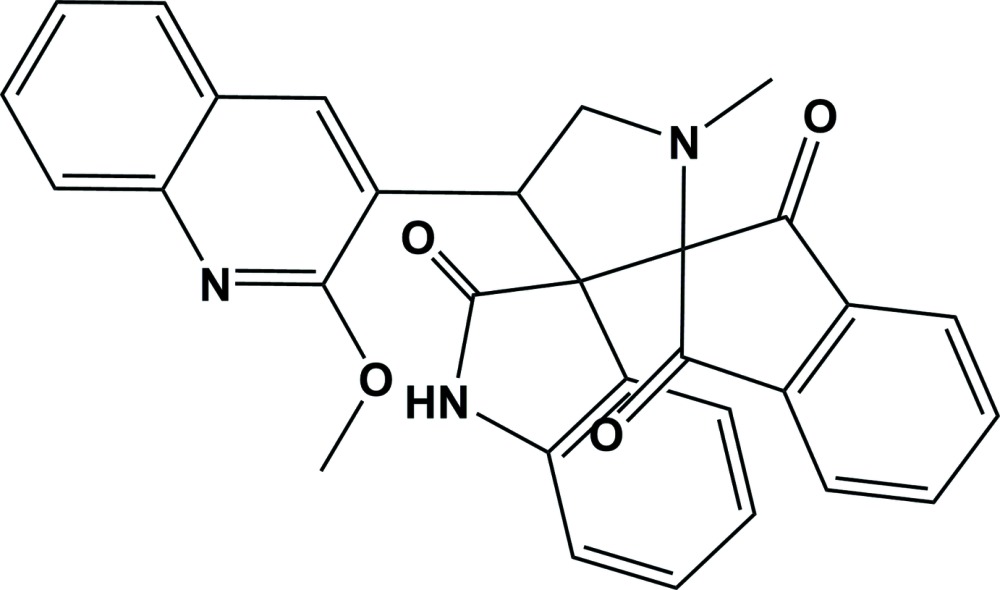


## Experimental   

### Crystal data  

C_30_H_23_N_3_O_4_
*M* *_r_* = 489.51
Monoclinic, 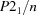

*a* = 10.9058 (3) Å
*b* = 9.5178 (5) Å
*c* = 23.8651 (6) Å
β = 95.378 (2)°
*V* = 2466.27 (16) Å^3^
*Z* = 4
Mo *K*α radiation
μ = 0.09 mm^−1^
*T* = 293 K
0.27 × 0.18 × 0.11 mm

### Data collection  

Bruker SMART APEXII area-detector diffractometer
Absorption correction: multi-scan (*SADABS*; Bruker, 2008 ▸) *T*_min_ = 0.976, *T*_max_ = 0.990
23642 measured reflections
6134 independent reflections
4376 reflections with *I* > 2σ(*I*)
*R*_int_ = 0.044

### Refinement  

*R*[*F* ^2^ > 2σ(*F* ^2^)] = 0.044
*wR*(*F* ^2^) = 0.115
*S* = 1.03
6134 reflections
337 parameters
H-atom parameters constrained
Δρ_max_ = 0.31 e Å^−3^
Δρ_min_ = −0.22 e Å^−3^

### 

Data collection: *APEX2* (Bruker, 2008 ▸); cell refinement: *SAINT* (Bruker, 2008 ▸); data reduction: *SAINT*; program(s) used to solve structure: *SHELXS97* (Sheldrick, 2008 ▸); program(s) used to refine structure: *SHELXL97* (Sheldrick, 2008 ▸); molecular graphics: *ORTEP-3 for Windows* (Farrugia, 2012 ▸) and *Mercury* (Macrae *et al.*, 2008 ▸); software used to prepare material for publication: *SHELXL97* and *PLATON* (Spek, 2009 ▸).

## Supplementary Material

Crystal structure: contains datablock(s) global, I. DOI: 10.1107/S2056989015023026/su5242sup1.cif

Structure factors: contains datablock(s) I. DOI: 10.1107/S2056989015023026/su5242Isup2.hkl

Supporting information file. DOI: 10.1107/S2056989015023026/su5242Isup3.cml

**Fig. 1.**
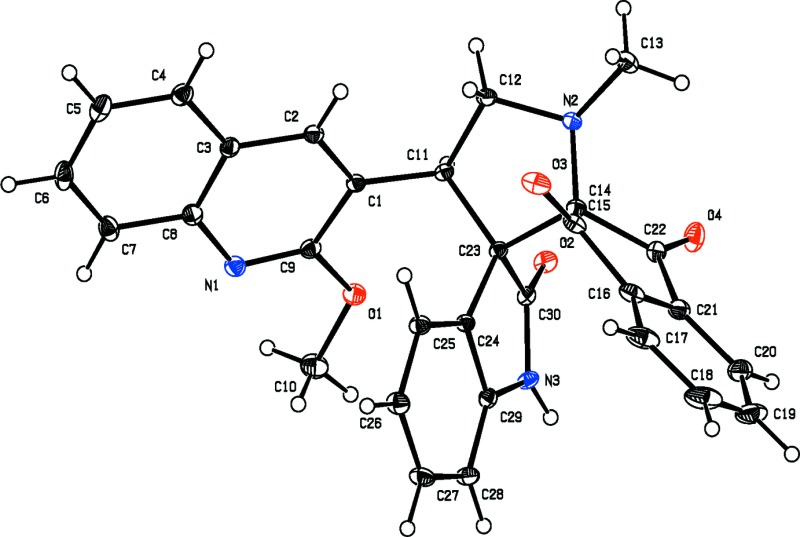
The molecular structure of the title compound, with atom labelling. Displacement ellipsoids are drawn at 30% probability level.

**Fig. 2.**
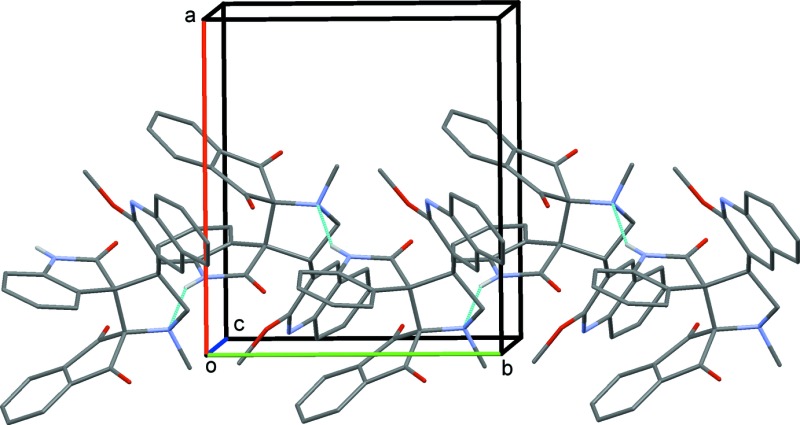
A partial view, along the c axis, of the crystal packing of the title compound, illustrating the formation of the hydrogen-bonded zigzag chains (dashed lines; see Table 1) running along the the b-axis direction. C-bound H atoms have been omitted for clarity.

**Fig. 3.**
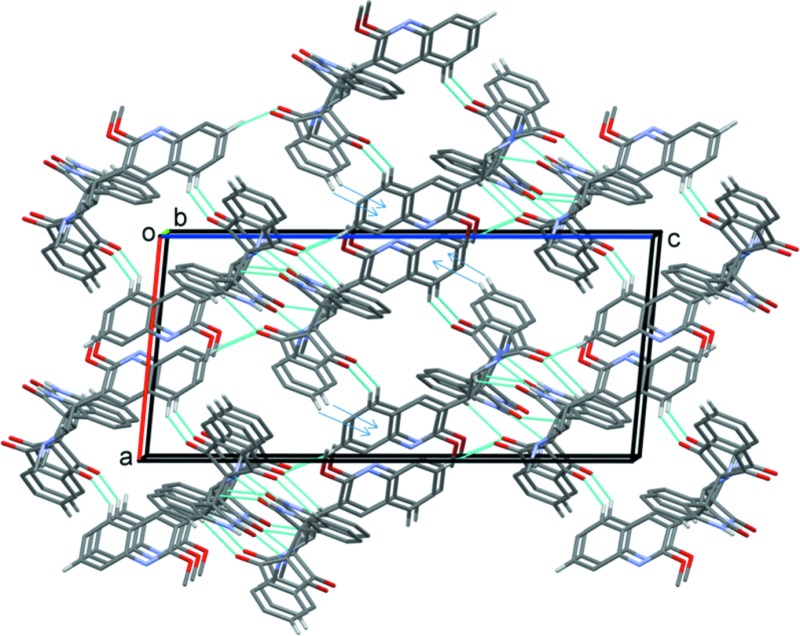
A view along the b axis of the crystal packing of the title compound. Hydrogen bonds are shown as dashed lines and C—H···π interactions as blue arrows (see Table 1). H atoms not involved in these interactions have been omitted for clarity.

. DOI: 10.1107/S2056989015023026/su5242fig1.tif
The mol­ecular structure of the title compound, with atom labelling. Displacement ellipsoids are drawn at 30% probability level.

c b- . DOI: 10.1107/S2056989015023026/su5242fig2.tif
A partial view, along the *c* axis, of the crystal packing of the title compound, illustrating the formation of the hydrogen-bonded zigzag chains (dashed lines; see Table 1) running along the the *b-*axis direction. C-bound H atoms have been omitted for clarity.

b . DOI: 10.1107/S2056989015023026/su5242fig3.tif
A view along the *b* axis of the crystal packing of the title compound. Hydrogen bonds are shown as dashed lines and C—H⋯π inter­actions as blue arrows (see Table 1). H atoms not involved in these inter­actions have been omitted for clarity.

CCDC reference: 1439764

Additional supporting information:  crystallographic information; 3D view; checkCIF report

## Figures and Tables

**Table 1:** Hydrogen-bond geometry (Å, °) *Cg*1 is the centroid of the C3–C8 ring.

*D*—H⋯*A*	*D*—H	H⋯*A*	*D*⋯*A*	*D*—H⋯*A*
N3—H3⋯N2^i^	0.86	2.19	2.971 (2)	151
C4—H4⋯O3^ii^	0.93	2.56	3.350 (2)	143
C6—H6⋯O4^iii^	0.93	2.42	3.307 (2)	159
C12—H12*A*⋯O2^iv^	0.97	2.53	3.325 (2)	139
C28—H28⋯O4^i^	0.93	2.56	3.354 (1)	144
C18—H18⋯*Cg*1^v^	0.93	2.89	3.778 (6)	160

Symmetry codes: (i) 
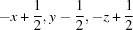
; (ii) 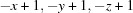; (iii) 
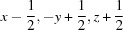
; (iv) 
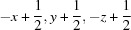
; (v) 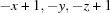.

## supplementary crystallographic information

### S1. Comment

The pyrrolidine ring system is found in a vast variety of compounds displaying an impressive range of biological activities (Babu *et al.*, 2012). Optically active pyrrolidines have been used as inter­mediates, chiral ligands or auxiliaries in controlled asymmetric synthesis (Savithri *et al.*, 2014). Pyrrolidine compounds are reported to exhibit anti­microbial, anti­fungal (Govind *et al.*, 2003), anti-influenza virus (Gayathri *et al.*, 2005), anti-inflammatory, anti­tumor (Li *et al.*, 2004), inhibit retroviral reverse transcriptases [i.e., human immunodeficiency virus type 1 (HIV-1)], cellular DNA polymerases, protein kinases (Bellina and Rossi, 2006), anti­biotics (Nirmala *et al.*, 2009), anti­convulsant, sphingosine-1-phosphate (S1P) receptor agonists, malic enzyme inhibitors, keto­amide-based cathepsin K inhibitors, human melanocortin-4 receptor agonists (Babu *et al.*, 2012). Indole compounds can be used as bioactive drugs. Indole derivatives exhibit anti­allergic, central nervous system depressant and muscle relaxant properties. In view of this biological importance, the title compound was synthesized and we report herein on its the crystal structure.

The molecular structure of the title compound is shown in Fig. 1. The central 1-methyl­pyrrolidine ring (N2/C11/C12/C14/C23) adopts a twist conformation on the N2—C12 bond. The pyrrolidine-2-one ring (N3/C23/C24/C29/C30) of the indoline-2-one ring system also has a twist conformation on the C23—C30 bond involving the spiro C atom and the carbonyl C atom. The five-membered ring (C14—C16/C21/C22) of the indene-1,3-dione moiety has an envelope conformation with atom C14 as the flap. The quinoline ring system adopts a planar conformation [r.m.s. deviation = 0.04 Å]. The mean planes of the indolin-2-one ring system, the indene-1,3-dione ring system and the the quinoline ring system are inclined to the mean plane of the central 1-methyl­pyrrolidine ring by 77.97 (7), 86.98 (7) and 46.58 (6) °, respectively.

In the crystal, molecules are linked *via* N—H···N hydrogen bonds forming zigzag chains along the *b* axis direction (Table 1 and Fig. 2). The chains are linked *via* number of C—H···O hydrogen bonds, and C—H···π and π-π inter­actions, involving inversion related quinoline units [Cg4···Cg5^i^ = 3.7404 (9) Å; where Cg4 and Cg5 are the centroids of rings N1/C1—C3/C8/C9 and C3—C8; symmetry code: (i) -x, -y+1, -z+1], forming a three-dimensional structure (Table 1 and Fig. 3).

### S2. Synthesis and crystallization

A mixture of indoline-2,3-dione (1 mmol) and 2-(methyl­amino)­acetic acid (1.5 mmol) were dissolved in methanol (100 ml) and refluxed for 5 min, followed by the addition of (*Z*)-3-((2-meth­oxy­quinolin-3-yl) methyl­ene) indolin-2-one (0.5 mmol), then the mixture was refluxed for 8 h. After completion of the reaction (monitored by silicagel precoated TLC), the title compound was separated from the cooled reaction mixture, filtered and dried under reduced pressure. Slow evaporation of a solution on the title compound in chloro­form/methanol (4:1) yielded light-yellow block-like crystals.

### S3. Refinement

Crystal data, data collection and structure refinement details are summarized in Table 2. The hydrogen atoms were placed in calculated positions and refined as riding atoms: C—H = 0.93-0.98 Å and N—H = 0.86 Å with *U*_iso_(H) = 1.5*U*_eq_(C-methyl) and 1.2*U*_eq_(N,C) for other H atoms.

### Crystal data

**Table:**

C_30_H_23_N_3_O_4_	*F*(000) = 1024
*M**_r_* = 489.51	*D*_x_ = 1.318 Mg m^−^^3^
Monoclinic, *P*2_1_/*n*	Mo *K*α radiation, λ = 0.71073 Å
Hall symbol: -P 2yn	Cell parameters from 6134 reflections
*a* = 10.9058 (3) Å	θ = 1.7–28.3°
*b* = 9.5178 (5) Å	µ = 0.09 mm^−^^1^
*c* = 23.8651 (6) Å	*T* = 293 K
β = 95.378 (2)°	Block, light yellow
*V* = 2466.27 (16) Å^3^	0.27 × 0.18 × 0.11 mm
*Z* = 4	

### Data collection

**Table:**

Bruker SMART APEXII area-detector diffractometer	6134 independent reflections
Radiation source: fine-focus sealed tube	4376 reflections with *I* > 2σ(*I*)
Graphite monochromator	*R*_int_ = 0.044
ω and φ scans	θ_max_ = 28.3°, θ_min_ = 1.7°
Absorption correction: multi-scan (*SADABS*; Bruker, 2008)	*h* = −14→14
*T*_min_ = 0.976, *T*_max_ = 0.990	*k* = −10→12
23642 measured reflections	*l* = −31→31

### Refinement

**Table:**

Refinement on *F*^2^	Secondary atom site location: difference Fourier map
Least-squares matrix: full	Hydrogen site location: inferred from neighbouring sites
*R*[*F*^2^ > 2σ(*F*^2^)] = 0.044	H-atom parameters constrained
*wR*(*F*^2^) = 0.115	*w* = 1/[σ^2^(*F*_o_^2^) + (0.0518*P*)^2^ + 0.5577*P*] where *P* = (*F*_o_^2^ + 2*F*_c_^2^)/3
*S* = 1.03	(Δ/σ)_max_ < 0.001
6134 reflections	Δρ_max_ = 0.31 e Å^−^^3^
337 parameters	Δρ_min_ = −0.22 e Å^−^^3^
0 restraints	Extinction correction: *SHELXL97* (Sheldrick, 2008), Fc^*^=kFc[1+0.001xFc^2^λ^3^/sin(2θ)]^-1/4^
Primary atom site location: structure-invariant direct methods	Extinction coefficient: 0.0015 (5)

### Special details

**Table:**

Geometry. All esds (except the esd in the dihedral angle between two l.s. planes) are estimated using the full covariance matrix. The cell esds are taken into account individually in the estimation of esds in distances, angles and torsion angles; correlations between esds in cell parameters are only used when they are defined by crystal symmetry. An approximate (isotropic) treatment of cell esds is used for estimating esds involving l.s. planes.
Refinement. Refinement of F^2^ against ALL reflections. The weighted R-factor wR and goodness of fit S are based on F^2^, conventional R-factors R are based on F, with F set to zero for negative F^2^. The threshold expression of F^2^ > 2sigma(F^2^) is used only for calculating R-factors(gt) etc. and is not relevant to the choice of reflections for refinement. R-factors based on F^2^ are statistically about twice as large as those based on F, and R- factors based on ALL data will be even larger.

### Fractional atomic coordinates and isotropic or equivalent isotropic displacement parameters (Å^2^)

**Table:**

	*x*	*y*	*z*	*U*_iso_*/*U*_eq_	
C1	0.20917 (13)	0.33633 (15)	0.40978 (6)	0.0154 (3)	
C2	0.25284 (13)	0.41678 (15)	0.45432 (6)	0.0164 (3)	
H2	0.3236	0.4701	0.4520	0.020*	
C3	0.19148 (13)	0.42037 (15)	0.50442 (6)	0.0176 (3)	
C4	0.22810 (14)	0.50968 (16)	0.55041 (6)	0.0214 (3)	
H4	0.2976	0.5660	0.5495	0.026*	
C5	0.16109 (15)	0.51327 (18)	0.59628 (6)	0.0250 (4)	
H5	0.1844	0.5736	0.6260	0.030*	
C6	0.05752 (15)	0.42652 (18)	0.59859 (6)	0.0261 (4)	
H6	0.0129	0.4296	0.6299	0.031*	
C7	0.02161 (14)	0.33733 (17)	0.55504 (6)	0.0233 (3)	
H7	−0.0459	0.2787	0.5574	0.028*	
C8	0.08663 (13)	0.33397 (16)	0.50662 (6)	0.0191 (3)	
C9	0.10099 (13)	0.25475 (16)	0.41718 (6)	0.0176 (3)	
C10	−0.04213 (16)	0.0851 (2)	0.37798 (8)	0.0343 (4)	
H10A	−0.0178	0.0186	0.4072	0.051*	
H10B	−0.0639	0.0360	0.3433	0.051*	
H10C	−0.1118	0.1378	0.3881	0.051*	
C11	0.26420 (13)	0.32928 (14)	0.35404 (5)	0.0145 (3)	
H11	0.1988	0.3560	0.3250	0.017*	
C12	0.37260 (13)	0.42704 (15)	0.34739 (6)	0.0169 (3)	
H12A	0.3448	0.5213	0.3372	0.020*	
H12B	0.4278	0.4311	0.3817	0.020*	
C13	0.54710 (14)	0.42826 (17)	0.28930 (6)	0.0228 (3)	
H13A	0.6039	0.4284	0.3226	0.034*	
H13B	0.5305	0.5232	0.2773	0.034*	
H13C	0.5823	0.3775	0.2599	0.034*	
C14	0.44003 (13)	0.21185 (15)	0.31502 (6)	0.0154 (3)	
C15	0.54468 (13)	0.16447 (16)	0.35943 (6)	0.0189 (3)	
C16	0.59196 (13)	0.02809 (16)	0.34030 (7)	0.0226 (3)	
C17	0.67272 (15)	−0.06517 (18)	0.36946 (8)	0.0333 (4)	
H17	0.7027	−0.0488	0.4067	0.040*	
C18	0.70682 (17)	−0.18363 (19)	0.34082 (10)	0.0431 (5)	
H18	0.7609	−0.2478	0.3592	0.052*	
C19	0.66172 (16)	−0.20855 (19)	0.28513 (10)	0.0402 (5)	
H19	0.6872	−0.2884	0.2670	0.048*	
C20	0.57999 (15)	−0.11731 (18)	0.25617 (8)	0.0307 (4)	
H20	0.5492	−0.1349	0.2192	0.037*	
C21	0.54552 (13)	0.00257 (17)	0.28481 (7)	0.0222 (3)	
C22	0.46541 (13)	0.12011 (16)	0.26393 (6)	0.0193 (3)	
C23	0.30953 (12)	0.17946 (15)	0.33795 (5)	0.0138 (3)	
C24	0.31584 (13)	0.06063 (15)	0.38042 (5)	0.0148 (3)	
C25	0.36072 (13)	0.05524 (16)	0.43670 (6)	0.0178 (3)	
H25	0.3945	0.1346	0.4549	0.021*	
C26	0.35408 (14)	−0.07186 (16)	0.46557 (6)	0.0218 (3)	
H26	0.3829	−0.0772	0.5034	0.026*	
C27	0.30461 (14)	−0.19003 (17)	0.43797 (6)	0.0236 (3)	
H27	0.3018	−0.2740	0.4578	0.028*	
C28	0.25907 (14)	−0.18660 (16)	0.38152 (6)	0.0211 (3)	
H28	0.2263	−0.2664	0.3633	0.025*	
C29	0.26467 (13)	−0.05929 (15)	0.35366 (5)	0.0162 (3)	
C30	0.22483 (13)	0.11534 (15)	0.28885 (5)	0.0156 (3)	
N1	0.04388 (11)	0.24888 (13)	0.46238 (5)	0.0198 (3)	
N2	0.43214 (11)	0.36056 (13)	0.30155 (5)	0.0161 (3)	
N3	0.21780 (11)	−0.02577 (13)	0.29860 (5)	0.0171 (3)	
H3	0.1884	−0.0862	0.2741	0.021*	
O1	0.05853 (9)	0.17940 (11)	0.37099 (4)	0.0226 (2)	
O2	0.17649 (9)	0.17942 (11)	0.24841 (4)	0.0201 (2)	
O3	0.58400 (10)	0.23063 (12)	0.40066 (4)	0.0262 (3)	
O4	0.42980 (10)	0.14597 (13)	0.21553 (4)	0.0287 (3)	

### Atomic displacement parameters (Å^2^)

**Table:**

	*U*^11^	*U*^22^	*U*^33^	*U*^12^	*U*^13^	*U*^23^
C1	0.0163 (7)	0.0128 (7)	0.0173 (6)	0.0012 (6)	0.0025 (5)	0.0014 (5)
C2	0.0178 (7)	0.0135 (7)	0.0179 (6)	−0.0001 (6)	0.0017 (5)	0.0010 (5)
C3	0.0208 (7)	0.0148 (8)	0.0173 (6)	0.0045 (6)	0.0027 (5)	0.0022 (6)
C4	0.0256 (8)	0.0183 (8)	0.0201 (7)	0.0041 (6)	0.0009 (6)	0.0004 (6)
C5	0.0320 (9)	0.0257 (9)	0.0169 (7)	0.0120 (7)	0.0002 (6)	−0.0002 (6)
C6	0.0296 (9)	0.0322 (10)	0.0177 (7)	0.0146 (7)	0.0075 (6)	0.0071 (7)
C7	0.0206 (8)	0.0257 (9)	0.0247 (7)	0.0063 (7)	0.0071 (6)	0.0076 (7)
C8	0.0194 (7)	0.0170 (8)	0.0210 (7)	0.0054 (6)	0.0029 (6)	0.0043 (6)
C9	0.0164 (7)	0.0147 (8)	0.0214 (7)	0.0014 (6)	0.0007 (5)	−0.0006 (6)
C10	0.0297 (9)	0.0328 (11)	0.0407 (10)	−0.0171 (8)	0.0054 (7)	−0.0098 (8)
C11	0.0166 (7)	0.0112 (7)	0.0153 (6)	0.0011 (6)	0.0001 (5)	−0.0002 (5)
C12	0.0210 (7)	0.0130 (7)	0.0166 (6)	−0.0021 (6)	0.0010 (5)	0.0001 (5)
C13	0.0219 (8)	0.0217 (8)	0.0252 (7)	−0.0012 (6)	0.0049 (6)	0.0068 (6)
C14	0.0175 (7)	0.0125 (7)	0.0160 (6)	0.0003 (6)	0.0008 (5)	0.0029 (5)
C15	0.0166 (7)	0.0188 (8)	0.0209 (7)	−0.0035 (6)	0.0000 (6)	0.0071 (6)
C16	0.0144 (7)	0.0175 (8)	0.0358 (9)	−0.0008 (6)	0.0021 (6)	0.0090 (7)
C17	0.0198 (8)	0.0235 (9)	0.0556 (11)	−0.0004 (7)	−0.0028 (8)	0.0167 (8)
C18	0.0225 (9)	0.0206 (10)	0.0853 (16)	0.0057 (8)	0.0009 (9)	0.0172 (10)
C19	0.0255 (9)	0.0152 (9)	0.0815 (15)	0.0024 (7)	0.0141 (9)	0.0000 (9)
C20	0.0227 (8)	0.0196 (9)	0.0518 (11)	−0.0001 (7)	0.0142 (8)	−0.0024 (8)
C21	0.0166 (7)	0.0168 (8)	0.0341 (8)	0.0009 (6)	0.0071 (6)	0.0027 (6)
C22	0.0189 (7)	0.0182 (8)	0.0214 (7)	0.0007 (6)	0.0051 (6)	−0.0002 (6)
C23	0.0159 (7)	0.0123 (7)	0.0129 (6)	0.0008 (6)	−0.0002 (5)	−0.0001 (5)
C24	0.0160 (7)	0.0121 (7)	0.0163 (6)	0.0011 (6)	0.0017 (5)	0.0015 (5)
C25	0.0208 (7)	0.0158 (8)	0.0162 (7)	−0.0014 (6)	−0.0016 (5)	−0.0006 (6)
C26	0.0270 (8)	0.0214 (8)	0.0162 (7)	−0.0009 (7)	−0.0018 (6)	0.0034 (6)
C27	0.0306 (8)	0.0160 (8)	0.0238 (8)	−0.0020 (7)	0.0008 (6)	0.0068 (6)
C28	0.0260 (8)	0.0137 (8)	0.0235 (7)	−0.0025 (6)	0.0010 (6)	−0.0003 (6)
C29	0.0173 (7)	0.0168 (8)	0.0145 (6)	0.0003 (6)	0.0014 (5)	−0.0012 (5)
C30	0.0167 (7)	0.0148 (7)	0.0153 (6)	0.0003 (6)	0.0012 (5)	−0.0016 (5)
N1	0.0187 (6)	0.0178 (7)	0.0232 (6)	0.0007 (5)	0.0036 (5)	0.0015 (5)
N2	0.0188 (6)	0.0135 (6)	0.0162 (6)	−0.0001 (5)	0.0028 (5)	0.0029 (5)
N3	0.0228 (6)	0.0126 (6)	0.0151 (6)	−0.0012 (5)	−0.0023 (5)	−0.0037 (5)
O1	0.0193 (5)	0.0224 (6)	0.0261 (5)	−0.0076 (5)	0.0019 (4)	−0.0057 (4)
O2	0.0247 (5)	0.0189 (6)	0.0153 (5)	0.0011 (4)	−0.0046 (4)	0.0009 (4)
O3	0.0282 (6)	0.0245 (6)	0.0240 (5)	−0.0073 (5)	−0.0076 (4)	0.0062 (5)
O4	0.0353 (6)	0.0339 (7)	0.0175 (5)	0.0083 (5)	0.0048 (5)	−0.0010 (5)

### Geometric parameters (Å, º)

**Table:**

C1—C2	1.3595 (19)	C14—C22	1.5455 (19)
C1—C9	1.4370 (19)	C14—C15	1.550 (2)
C1—C11	1.5112 (18)	C14—C23	1.6019 (18)
C2—C3	1.4243 (18)	C15—O3	1.2122 (18)
C2—H2	0.9300	C15—C16	1.484 (2)
C3—C8	1.413 (2)	C16—C17	1.390 (2)
C3—C4	1.416 (2)	C16—C21	1.394 (2)
C4—C5	1.373 (2)	C17—C18	1.387 (3)
C4—H4	0.9300	C17—H17	0.9300
C5—C6	1.404 (2)	C18—C19	1.394 (3)
C5—H5	0.9300	C18—H18	0.9300
C6—C7	1.370 (2)	C19—C20	1.382 (3)
C6—H6	0.9300	C19—H19	0.9300
C7—C8	1.4117 (19)	C20—C21	1.399 (2)
C7—H7	0.9300	C20—H20	0.9300
C8—N1	1.3768 (19)	C21—C22	1.477 (2)
C9—N1	1.2965 (18)	C22—O4	1.2091 (17)
C9—O1	1.3595 (17)	C23—C24	1.5159 (19)
C10—O1	1.4398 (19)	C23—C30	1.5478 (19)
C10—H10A	0.9600	C24—C25	1.3867 (18)
C10—H10B	0.9600	C24—C29	1.398 (2)
C10—H10C	0.9600	C25—C26	1.397 (2)
C11—C12	1.5244 (19)	C25—H25	0.9300
C11—C23	1.5686 (19)	C26—C27	1.387 (2)
C11—H11	0.9800	C26—H26	0.9300
C12—N2	1.4668 (17)	C27—C28	1.392 (2)
C12—H12A	0.9700	C27—H27	0.9300
C12—H12B	0.9700	C28—C29	1.386 (2)
C13—N2	1.4633 (18)	C28—H28	0.9300
C13—H13A	0.9600	C29—N3	1.4013 (17)
C13—H13B	0.9600	C30—O2	1.2191 (16)
C13—H13C	0.9600	C30—N3	1.3664 (19)
C14—N2	1.4521 (18)	N3—H3	0.8600
			
C2—C1—C9	116.09 (12)	O3—C15—C14	125.85 (14)
C2—C1—C11	125.05 (13)	C16—C15—C14	107.38 (12)
C9—C1—C11	118.85 (12)	C17—C16—C21	121.39 (16)
C1—C2—C3	120.81 (13)	C17—C16—C15	128.78 (15)
C1—C2—H2	119.6	C21—C16—C15	109.81 (13)
C3—C2—H2	119.6	C16—C17—C18	117.31 (18)
C8—C3—C4	119.40 (13)	C16—C17—H17	121.3
C8—C3—C2	117.56 (13)	C18—C17—H17	121.3
C4—C3—C2	123.00 (14)	C17—C18—C19	121.42 (17)
C5—C4—C3	120.05 (15)	C17—C18—H18	119.3
C5—C4—H4	120.0	C19—C18—H18	119.3
C3—C4—H4	120.0	C20—C19—C18	121.55 (17)
C4—C5—C6	120.47 (15)	C20—C19—H19	119.2
C4—C5—H5	119.8	C18—C19—H19	119.2
C6—C5—H5	119.8	C19—C20—C21	117.27 (17)
C7—C6—C5	120.54 (14)	C19—C20—H20	121.4
C7—C6—H6	119.7	C21—C20—H20	121.4
C5—C6—H6	119.7	C16—C21—C20	121.04 (15)
C6—C7—C8	120.34 (15)	C16—C21—C22	109.82 (13)
C6—C7—H7	119.8	C20—C21—C22	129.08 (15)
C8—C7—H7	119.8	O4—C22—C21	127.10 (14)
N1—C8—C3	122.06 (12)	O4—C22—C14	124.99 (14)
N1—C8—C7	118.75 (14)	C21—C22—C14	107.81 (12)
C3—C8—C7	119.17 (14)	C24—C23—C30	101.44 (11)
N1—C9—O1	119.89 (13)	C24—C23—C11	120.68 (11)
N1—C9—C1	126.12 (13)	C30—C23—C11	111.36 (11)
O1—C9—C1	113.99 (12)	C24—C23—C14	112.70 (11)
O1—C10—H10A	109.5	C30—C23—C14	107.67 (10)
O1—C10—H10B	109.5	C11—C23—C14	102.66 (11)
H10A—C10—H10B	109.5	C25—C24—C29	120.11 (13)
O1—C10—H10C	109.5	C25—C24—C23	131.68 (13)
H10A—C10—H10C	109.5	C29—C24—C23	108.21 (11)
H10B—C10—H10C	109.5	C24—C25—C26	118.66 (13)
C1—C11—C12	116.13 (11)	C24—C25—H25	120.7
C1—C11—C23	114.59 (11)	C26—C25—H25	120.7
C12—C11—C23	105.31 (11)	C27—C26—C25	120.25 (13)
C1—C11—H11	106.7	C27—C26—H26	119.9
C12—C11—H11	106.7	C25—C26—H26	119.9
C23—C11—H11	106.7	C26—C27—C28	121.89 (14)
N2—C12—C11	102.48 (11)	C26—C27—H27	119.1
N2—C12—H12A	111.3	C28—C27—H27	119.1
C11—C12—H12A	111.3	C29—C28—C27	117.19 (14)
N2—C12—H12B	111.3	C29—C28—H28	121.4
C11—C12—H12B	111.3	C27—C28—H28	121.4
H12A—C12—H12B	109.2	C28—C29—C24	121.88 (13)
N2—C13—H13A	109.5	C28—C29—N3	128.32 (13)
N2—C13—H13B	109.5	C24—C29—N3	109.71 (12)
H13A—C13—H13B	109.5	O2—C30—N3	126.84 (13)
N2—C13—H13C	109.5	O2—C30—C23	125.82 (13)
H13A—C13—H13C	109.5	N3—C30—C23	107.32 (11)
H13B—C13—H13C	109.5	C9—N1—C8	117.27 (13)
N2—C14—C22	112.79 (11)	C14—N2—C13	116.06 (11)
N2—C14—C15	117.35 (12)	C14—N2—C12	106.06 (10)
C22—C14—C15	101.55 (11)	C13—N2—C12	113.99 (12)
N2—C14—C23	103.12 (11)	C30—N3—C29	111.23 (11)
C22—C14—C23	113.03 (11)	C30—N3—H3	124.4
C15—C14—C23	109.34 (10)	C29—N3—H3	124.4
O3—C15—C16	126.68 (14)	C9—O1—C10	116.13 (12)
			
C9—C1—C2—C3	−1.3 (2)	C1—C11—C23—C24	−9.78 (18)
C11—C1—C2—C3	177.52 (13)	C12—C11—C23—C24	119.09 (13)
C1—C2—C3—C8	2.1 (2)	C1—C11—C23—C30	108.89 (13)
C1—C2—C3—C4	−175.45 (14)	C12—C11—C23—C30	−122.25 (12)
C8—C3—C4—C5	−0.9 (2)	C1—C11—C23—C14	−136.15 (12)
C2—C3—C4—C5	176.61 (14)	C12—C11—C23—C14	−7.29 (13)
C3—C4—C5—C6	1.4 (2)	N2—C14—C23—C24	−150.97 (11)
C4—C5—C6—C7	−0.2 (2)	C22—C14—C23—C24	86.94 (14)
C5—C6—C7—C8	−1.5 (2)	C15—C14—C23—C24	−25.39 (15)
C4—C3—C8—N1	177.54 (13)	N2—C14—C23—C30	97.99 (12)
C2—C3—C8—N1	−0.1 (2)	C22—C14—C23—C30	−24.10 (15)
C4—C3—C8—C7	−0.8 (2)	C15—C14—C23—C30	−136.42 (12)
C2—C3—C8—C7	−178.43 (13)	N2—C14—C23—C11	−19.61 (12)
C6—C7—C8—N1	−176.40 (14)	C22—C14—C23—C11	−141.71 (11)
C6—C7—C8—C3	2.0 (2)	C15—C14—C23—C11	105.97 (12)
C2—C1—C9—N1	−1.6 (2)	C30—C23—C24—C25	−168.81 (14)
C11—C1—C9—N1	179.44 (14)	C11—C23—C24—C25	−45.3 (2)
C2—C1—C9—O1	178.08 (12)	C14—C23—C24—C25	76.33 (18)
C11—C1—C9—O1	−0.85 (19)	C30—C23—C24—C29	11.01 (14)
C2—C1—C11—C12	−3.5 (2)	C11—C23—C24—C29	134.53 (13)
C9—C1—C11—C12	175.32 (12)	C14—C23—C24—C29	−103.85 (13)
C2—C1—C11—C23	119.73 (15)	C29—C24—C25—C26	0.3 (2)
C9—C1—C11—C23	−61.45 (17)	C23—C24—C25—C26	−179.91 (14)
C1—C11—C12—N2	159.38 (11)	C24—C25—C26—C27	0.7 (2)
C23—C11—C12—N2	31.44 (13)	C25—C26—C27—C28	−0.7 (2)
N2—C14—C15—O3	35.42 (19)	C26—C27—C28—C29	−0.2 (2)
C22—C14—C15—O3	158.85 (14)	C27—C28—C29—C24	1.2 (2)
C23—C14—C15—O3	−81.49 (17)	C27—C28—C29—N3	−175.11 (14)
N2—C14—C15—C16	−141.36 (12)	C25—C24—C29—C28	−1.3 (2)
C22—C14—C15—C16	−17.93 (14)	C23—C24—C29—C28	178.90 (13)
C23—C14—C15—C16	101.74 (12)	C25—C24—C29—N3	175.66 (12)
O3—C15—C16—C17	13.5 (3)	C23—C24—C29—N3	−4.18 (15)
C14—C15—C16—C17	−169.79 (15)	C24—C23—C30—O2	167.42 (13)
O3—C15—C16—C21	−164.90 (14)	C11—C23—C30—O2	37.77 (18)
C14—C15—C16—C21	11.84 (15)	C14—C23—C30—O2	−74.05 (16)
C21—C16—C17—C18	1.0 (2)	C24—C23—C30—N3	−14.36 (13)
C15—C16—C17—C18	−177.20 (15)	C11—C23—C30—N3	−144.01 (11)
C16—C17—C18—C19	−0.2 (3)	C14—C23—C30—N3	104.18 (12)
C17—C18—C19—C20	−0.8 (3)	O1—C9—N1—C8	−176.13 (12)
C18—C19—C20—C21	0.9 (2)	C1—C9—N1—C8	3.6 (2)
C17—C16—C21—C20	−0.9 (2)	C3—C8—N1—C9	−2.6 (2)
C15—C16—C21—C20	177.64 (13)	C7—C8—N1—C9	175.76 (14)
C17—C16—C21—C22	−178.35 (14)	C22—C14—N2—C13	−68.82 (15)
C15—C16—C21—C22	0.15 (16)	C15—C14—N2—C13	48.69 (16)
C19—C20—C21—C16	−0.1 (2)	C23—C14—N2—C13	168.92 (11)
C19—C20—C21—C22	176.86 (15)	C22—C14—N2—C12	163.46 (11)
C16—C21—C22—O4	164.54 (15)	C15—C14—N2—C12	−79.03 (14)
C20—C21—C22—O4	−12.7 (3)	C23—C14—N2—C12	41.21 (13)
C16—C21—C22—C14	−12.15 (16)	C11—C12—N2—C14	−46.38 (13)
C20—C21—C22—C14	170.62 (15)	C11—C12—N2—C13	−175.31 (11)
N2—C14—C22—O4	−32.2 (2)	O2—C30—N3—C29	−168.83 (13)
C15—C14—C22—O4	−158.68 (14)	C23—C30—N3—C29	12.97 (15)
C23—C14—C22—O4	84.30 (18)	C28—C29—N3—C30	170.86 (14)
N2—C14—C22—C21	144.58 (12)	C24—C29—N3—C30	−5.81 (16)
C15—C14—C22—C21	18.10 (14)	N1—C9—O1—C10	−6.3 (2)
C23—C14—C22—C21	−98.91 (13)	C1—C9—O1—C10	173.97 (13)

### Hydrogen-bond geometry (Å, º)

Cg1 is the centroid of the C3–C8 ring.

**Table:**

*D*—H···*A*	*D*—H	H···*A*	*D*···*A*	*D*—H···*A*
N3—H3···N2^i^	0.86	2.19	2.971 (2)	151
C4—H4···O3^ii^	0.93	2.56	3.350 (2)	143
C6—H6···O4^iii^	0.93	2.42	3.307 (2)	159
C12—H12*A*···O2^iv^	0.97	2.53	3.325 (2)	139
C28—H28···O4^i^	0.93	2.56	3.354 (1)	144
C18—H18···*Cg*1^v^	0.93	2.89	3.778 (6)	160

Symmetry codes: (i) −*x*+1/2, *y*−1/2, −*z*+1/2; (ii) −*x*+1, −*y*+1, −*z*+1; (iii) *x*−1/2, −*y*+1/2, *z*+1/2; (iv) −*x*+1/2, *y*+1/2, −*z*+1/2; (v) −*x*+1, −*y*, −*z*+1.
