# Supplementary figures and images for: Crystal structure of 4′-(2-meth­oxy­quinolin-3-yl)-1′-methyl­dispiro­[indan-2,2′-pyrrolidine-3′,3′′-indoline]-1,3,2′′-trione
Source: Acta Crystallogr E Crystallogr Commun. 2015 Dec 12;71(Pt 12):o1038–9. doi: 10.1107/S2056989015023026 (PMC4719967; doi:10.1107/S2056989015023026)

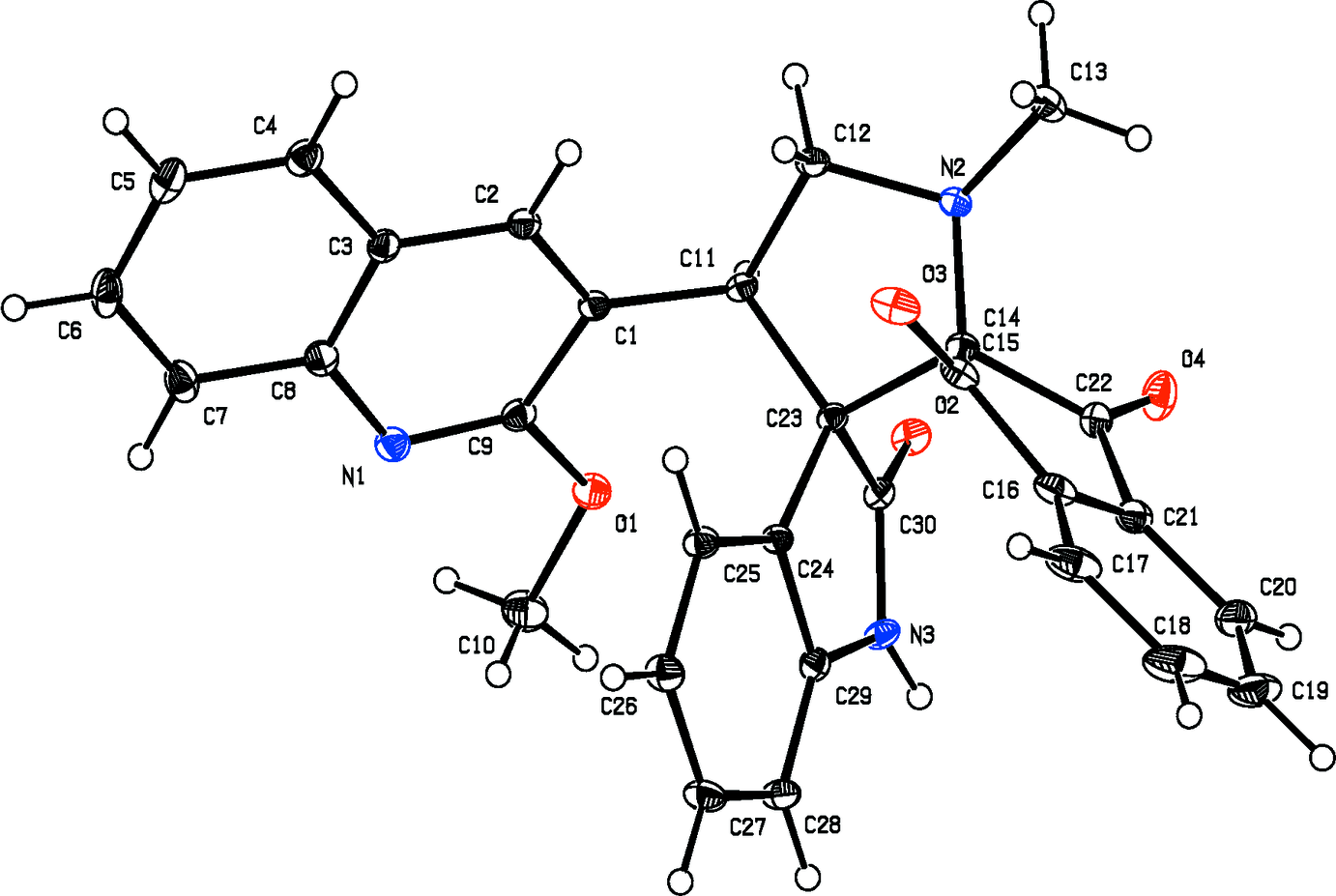

Supplement: Supplementary file 4 [file e-71-o1038-fig1.tif]

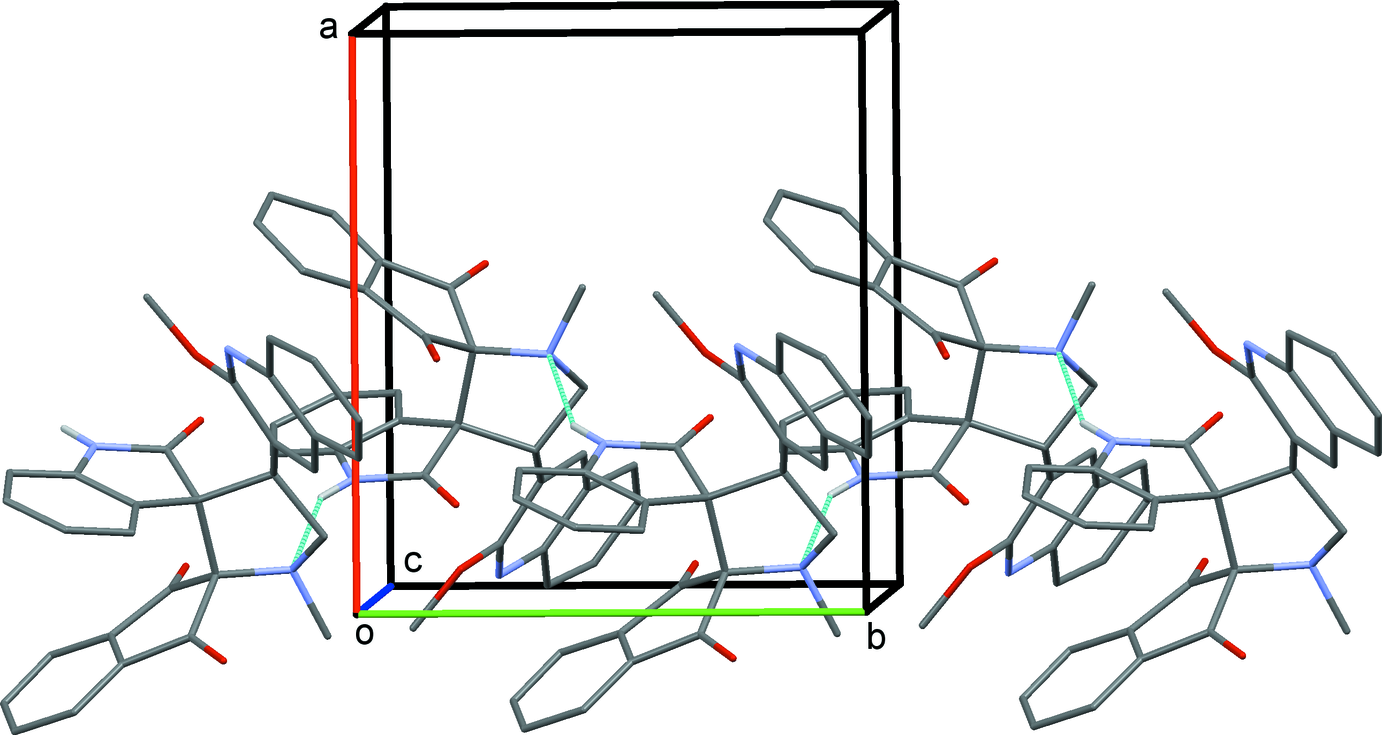

Supplement: Supplementary file 5 [file e-71-o1038-fig2.tif]

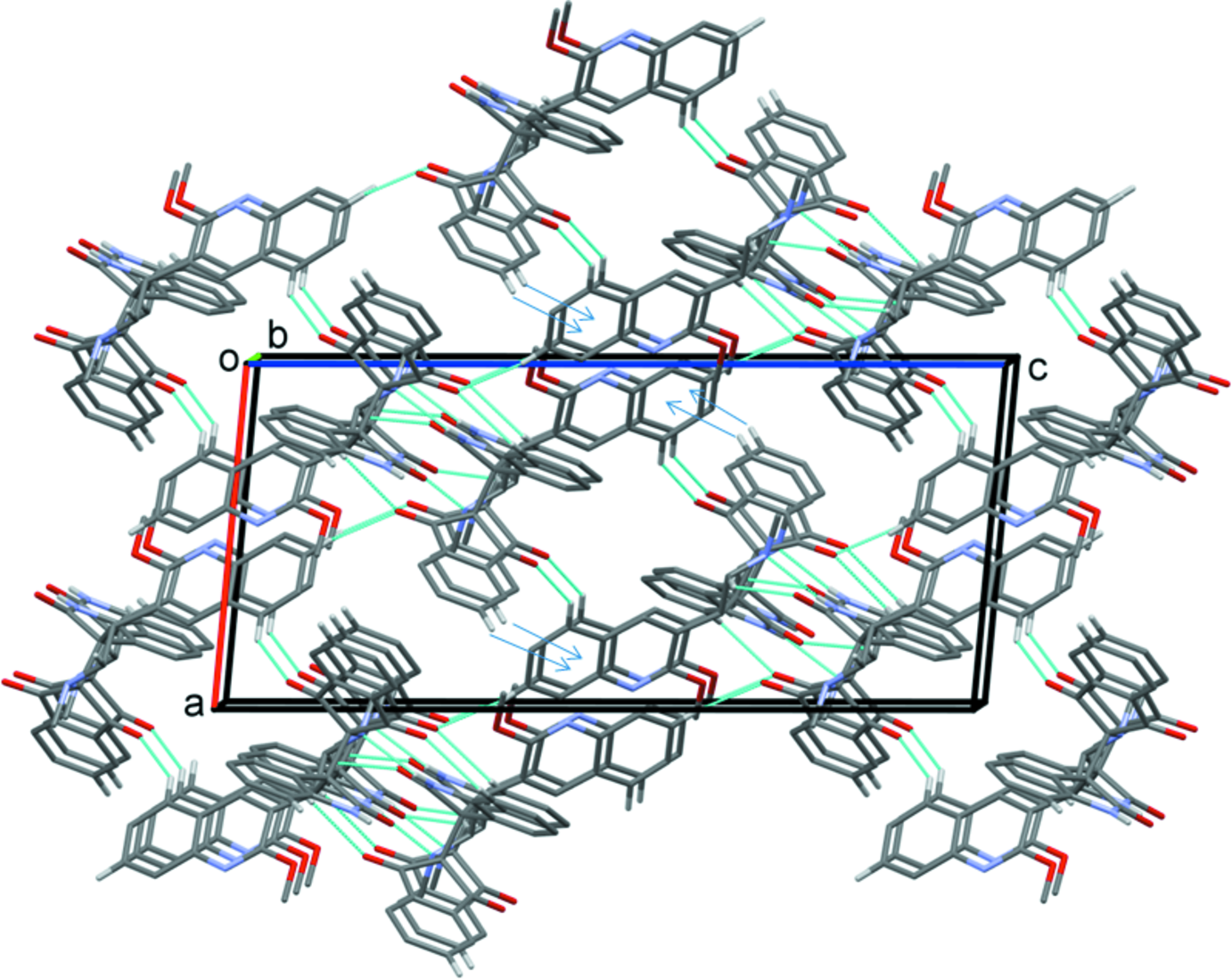

Supplement: Supplementary file 6 [file e-71-o1038-fig3.tif]
